# The Antiviral Restriction Factors IFITM1, 2 and 3 Do Not Inhibit Infection of Human Papillomavirus, Cytomegalovirus and Adenovirus

**DOI:** 10.1371/journal.pone.0096579

**Published:** 2014-05-14

**Authors:** Cody J. Warren, Laura M. Griffin, Alexander S. Little, I-Chueh Huang, Michael Farzan, Dohun Pyeon

**Affiliations:** 1 Department of Microbiology, University of Colorado School of Medicine, Aurora, Colorado, United States of America; 2 Department of Medicine, University of Colorado School of Medicine, Aurora, Colorado, United States of America; 3 Department of Cell Biology and Neuroscience, University of California Riverside, Riverside, California, United States of America; 4 Department of Infectious Diseases, The Scripps Research Institute, Jupiter, Florida, United States of America; University of Hong Kong, Hong Kong

## Abstract

Type I interferons (IFN-α and β) induce dynamic host defense mechanisms to inhibit viral infections. It has been recently recognized that the interferon-inducible transmembrane proteins (IFITM) 1, 2 and 3 can block entry of a broad spectrum of RNA viruses. However, no study to date has focused on the role of IFITM proteins in DNA virus restriction. Here, we demonstrate that IFN-α or -β treatment of keratinocytes substantially decreases human papillomavirus 16 (HPV16) infection while robustly inducing IFITM1, 2 and 3 expression. However, IFITM1, 2 and 3 overexpression did not inhibit HPV16 infection; rather, IFITM1 and IFITM3 modestly enhanced HPV16 infection in various cell types including primary keratinocytes. Moreover, IFITM1, 2 and 3 did not inhibit infection by two other DNA viruses, human cytomegalovirus (HCMV) and adenovirus type 5 (Ad5). Taken together, we reveal that the entry of several DNA viruses, including HPV, HCMV, and Ad5 is not affected by IFITM1, 2 and 3 expression. These results imply that HPV, and other DNA viruses, may bypass IFITM restriction during intracellular trafficking.

## Introduction

Human papillomaviruses (HPVs) are small, non-enveloped double-stranded DNA viruses causally associated with multiple human cancers. Over 170 different genotypes have been identified and collectively categorized into high-risk or low-risk genotypes depending on their oncogenic capacity [1], [2]. The high-risk types are most commonly associated with cervical cancer [3], [4] and increasing evidence points to a contributing role in other cancers including head-neck [5], [6] and anogenital cancers [7]. HPV16 is the most prevalent high-risk genotype and serves as the main vaccine target along with HPV18 [8], [9].

HPV is the most common sexually transmitted pathogen in the United States [10]. Despite high exposure rates, most people clear their infections naturally within 1–2 years [11]. However, long-term persistent infections are established in approximately 10% of women [12]. Since persistence is required for cancer progression [13], [14], it is critical to understand host immune features that are responsible for viral clearance so that new approaches targeting persistent HPV infections can be developed.

In order to establish long-term infections, HPVs must actively avoid both adaptive and innate immune responses. HPVs prevent adaptive immune detection by several mechanisms in their unique life cycle. First, viral antigen production is limited to terminally differentiated keratinocytes of the mucosal and cutaneous epithelia. These cells are programmed to die of terminal differentiation, thus virus release coincides with limited inflammation and release of danger signals [15]. Additionally, there is no viremic stage of the HPV life cycle, which minimizes the activation of systemic immune responses [16]. Despite eliciting weak adaptive immune responses, the majority of primary HPV infections are cleared, thus suggesting the involvement of additional immune-mediated control mechanisms. Keratinocytes intrinsically express low-levels of interferons (IFNs) α, β, and κ which induce interferon-stimulated gene (ISG) expression [17], [18]. However, the HPV oncoproteins E6 and E7 actively target the IFN regulatory transcription factors IRF-3 and IRF-1, respectively, resulting in an overall dampening of ISG responses during infection [19]–[21]. Active subversion of the IFN pathway suggests that the innate immune response, specifically IFN-regulated genes, may interfere with HPV persistence.

The IFN-inducible transmembrane (IFITM) proteins are a family of ubiquitously expressed restriction factors that mediate IFN-induced antiviral activity [22], [23]. The antiviral effects of IFITMs were first discovered in a genetic screen for host factors that restrict influenza A virus replication [24]. Follow-up studies revealed IFITM type-specific restriction of an array of RNA viruses including Marburg and Ebola filoviruses (MARV, EBOV), dengue and West Nile (WNV) flaviviruses, SARS coronavirus (SARS-CoV), hepatitis C virus (HCV), human immunodeficiency virus (HIV), Rift Valley fever virus (RVFV), respiratory syncytial virus (RSV), and reovirus [25]–[30]. One of the common entry mechanisms shared by all these viruses, except HIV, is the requirement for low pH in late endosomes or lysosomes to facilitate genome release into the cytosol [22], [23].

HPVs encapsidate their 8 kb, double-stranded DNA genome, in a desiccant-resistant icosahedral capsid composed of the major and minor capsid proteins L1 and L2, respectively [31]. Devoid of an envelope, initial cell contact is mediated at the basement membrane of epithelial cells by direct binding of the L1 capsid protein to heparan sulfate proteoglycans [32]. While downstream events in entry are not yet fully elucidated, it is well known that uncoating and genome translocation to the nucleus is dependent on the low pH encountered in acidified late endosomes and lysosomes [33]–[35]. As the antiviral activity of IFITMs is likely mediated by preventing endosome fusion and viral entry into the cytosol [22], [23], [36], we hypothesized that one or more types of IFITM proteins inhibit HPV entry by preventing escape from the endocytic pathway.

Our study demonstrates that type I IFNs inhibit HPV infection in keratinocytes, thus suggesting that ISG induction may be critical for HPV restriction. Surprisingly, overexpression of IFITM1 and IFITM3 consistently enhanced HPV infectivity in various epithelial cell lines and keratinocytes, while knockdown of endogenous IFITMs yielded no effect. Additionally, two other DNA viruses, HCMV and Ad5, were unaffected by IFITM overexpression. Analysis of the rate of HPV capsid protein degradation in IFITM1 overexpressing primary keratinocytes revealed a delay in proteolytic degradation of virus capsids. Collectively, our results suggest that endosome trafficking, altered by IFITM overexpression, preferentially routes HPV to a more productive infectious pathway. Based on these results, we present the first study detailing the role of IFITMs in the entry of DNA viruses, showing that IFITM1, 2 and 3 proteins do not restrict HPV, HCMV, and Ad5 infections. Our results suggest an evolutionarily conserved entry mechanism by these DNA viruses that bypasses the antiviral function of IFITMs that restrict many RNA viruses.

## Materials and Methods

### Viruses and Reagents

HPV16 virions and pseudovirions were prepared as described previously [37], [38]. pLucF, used in generating HPV16-LucF pseudovirions, was a kind gift from Chris Buck. Adenovirus type 5 (Ad5-CMV-GFP), human cytomegalovirus (HCMV TB40E mCherry) and SARS-CoV pseudotyped lentivirus [39] were generous gifts from Drs. Jerome Schaack, Caroline Kulesza, and Kathryn Holmes, respectively (University of Colorado School of Medicine). Human leukocyte IFN-α (NR-3078) and human recombinant IFN-β (NR-3085) were obtained from BEI Resources (Manassas, VA).

### Cell Lines

293FT cells purchased from Invitrogen (Grand Island, NY) and HeLa cells obtained from Dr. Paul Lambert (University of Wisconsin-Madison) were maintained in Dulbecco’s modified eagle’s medium (DMEM) (Thermo Scientific/HyClone, Logan, UT) supplemented with 10% fetal bovine serum (FBS) (HyClone). HaCaT cells [40] were also obtained from Dr. Paul Lambert and were cultured in E-medium (3 parts DMEM, 1 part Ham’s F-12 nutrient mixture) supplemented with 5% FBS. Human foreskin keratinocytes (HFKs), derived from 3 neonatal foreskin donors, were purchased from Invitrogen (Cascade Biologics, Portland, OR) and cultured in EpiLife medium with 60 µM calcium supplemented with human keratinocyte growth supplement (Invitrogen/Cascade Biologics) according to the manufacturer’s protocol [41].

Human lung epithelial A549 cells transduced with vector alone (pQCXIP) or stably expressing c-Myc tagged IFITM1, 2, or 3 were generated previously [25] and maintained in Roswell Park Memorial Institute (RPMI) 1640 (HyClone). HeLa cell lines stably expressing pRS vector-based control shRNA and shRNA targeting IFITM1 or 3 [25] were maintained as described above. HeLa and HaCaT cell lines transfected with vector alone or stably expressing IFITM proteins were selected with 3 µg/mL of puromycin (Invitrogen).

### Production of Retroviruses for Transduction

To produce transducing retroviruses, 293FT cells were transfected using the PEI method with a 1∶3∶4 DNA ratio of pCMV-VSV-G, pMLV gag-pol (Dr. Jerome Schaack) and transfer vector, respectively. After 48 and 72 h, culture supernatants were combined, passed through a 0.45 µm syringe filter and concentrated 100 times by centrifugation at 25,000 rpm for 3 h at 4°C.

### Infection Assays

HPV16-LucF and SARS-CoV pseudovirion infectivity were measured by luciferase assay as described previously [39], [41]. Infections with HPV16-LucF pesudovirions were performed at 10 to 10,000 viral genome equivalents (vge)/cell, which is equivalent to approximately 0.3 to 300 multiplicity of infection (MOI). GFP and phycoerythrin (PE) signals from cells infected with Ad5-CMV-GFP and HCMV TB40E mCherry, respectively, were collected from at least 20,000 cells using a FACScalibur flow cytometer (BD Bioscience). Data were analyzed using FlowJo software (Tree Star).

### Western Blot

Cells were lysed in RIPA buffer containing protease inhibitor cocktail (Roche) and 20 µg of total protein was separated by SDS-PAGE. Antibodies: Mouse monoclonal anti-L1 (1∶1000, CAMVIR-1, Abcam), mouse monoclonal anti-c-Myc (1∶1000, 9E10, Santa Cruz Biotechnology), mouse monoclonal anti-β-actin (1∶5000, 8H10D10, Cell Signaling Technology), rabbit polyclonal anti-IFITM1 (1∶1000, 11727-3-AP, Proteintech), rabbit polyclonal anti-IFITM3 (1∶500, H00010410-D03P, Novus Biologicals) and HRP-conjugated secondary antibodies (1∶10,000, Jackson ImmunoResearch Laboratories). Proteins were visualized using Clarity Western ECL substrate (Bio-Rad) and the ChemiDoc XRS System (Bio-Rad).

### Reverse Transcription-quantitative PCR (RT-qPCR)

Total RNA was extracted using RNeasy kit (Qiagen) and cDNA was synthesized using oligo (dT) and Superscript II reverse transcriptase (Invitrogen). cDNA was analyzed by quantitative PCR (qPCR) using Fast Start Universal SYBR Green Master Mix (Roche). Primers specific for selected ISGs (Table S1) were designed using Primer-BLAST (http://www.ncbi.nlm.nih.gov/tools/primer-blast/) and β-actin sequences have been described previously [42]. qPCR was performed using CFX Connect Real-Time PCR Detection System (Bio-Rad). Using the 2^−ΔΔCT^ method, relative quantities were calculated and normalized to β-actin.

## Results

### Type I IFNs Efficiently Inhibit Infection of HPV16 in Human Keratinocytes

**Figure 1:**
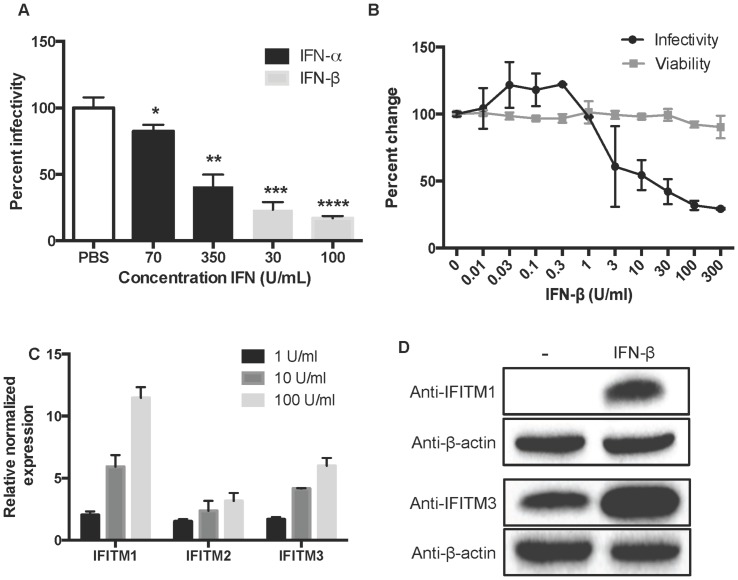
Type I IFNs efficiently inhibit HPV16 entry into human keratinocytes. Human keratinocyte HaCaT cells were inoculated with HPV16-LucF pseudovirions after 24 h pre-treatment with (A) IFN-α, IFN-β, or vehicle (PBS) or (B) the indicated concentrations of IFN-β. The reporter luciferase activity (A & B) and cell viability (B) were measured by Bright-Glo Luciferase Assay System (Promega) and CellTiter-Glo Luminescent Cell Viability Assay (Promega), respectively. Mean % infectivity and % viability compared to untreated cells with standard deviations are shown. *P*-values were calculated by Student’s two-tailed *t*-test using Prism version 6.0 for Mac, GraphPad software (San Diego California USA, www.graphpad.com). Significant differences (*p < 0.05, **p < 0.01, ***p < 0.001 and ****p < 0.0001) compared to PBS treated cells are marked with asterisks. (C) IFITM1, 2 and 3 expression levels were determined by RT-qPCR using IFITM specific primers (Table S1). Data are presented as fold change compared to untreated cells. (D) HaCaT cells were treated with 100 U/ml of IFN-β or medium alone and endogenous IFITM1 or IFITM3 protein expression in cell lysate was analyzed by western blotting using specific antibodies. PageRuler plus ladder (Thermo) was used to approximate molecular weights in kilodaltons (kD): IFITM1, 15 kD; IFITM3, 17 kD; β-actin, 46 kD.

To determine whether type I IFNs interfere with HPV16 infection, HaCaT keratinocytes were pre-treated with IFN-α or IFN-β for 24 h and then infected with HPV16 luciferase reporter pseudovirions (HPV16-LucF) in the presence of IFN-α or IFN-β. At 48 h post infection (hpi), infectivity was measured by relative luciferase activity [43]. Although both IFN-α and –β treatments significantly inhibited HPV16-LucF infection, the reductions seen with IFN-β treatment were more robust at lower doses (Fig. 1A). RT-qPCR of selected ISGs (MX1, IFI44, IFIT1, IFITM1, 2, and 3) revealed enhanced expression of mRNAs following IFN-α treatment (Fig. S1). This indicates that, although keratinocytes are responsive to IFN-α stimulation, IFN-β treatment is more effective at reducing infectivity despite sharing a common surface receptor [44]. To exclude the possibility that the observed effect of IFN-β on HPV infection might reflect cytotoxicity rather than inhibiting infection, we measured cell viability using a luminescence-based ATP quantification assay in parallel (Promega). No significant effect on keratinocyte viability was observed up to 300 U/ml of IFN-β (Fig. 1B).

IFITM protein expression induced by type I IFN inhibits infection of many RNA viruses [22]–[30]. Therefore, we analyzed induction of IFITM1, 2 and 3 expression by IFN-β treatment in human keratinocytes, the natural host cells for HPV. HaCaT cells were treated with increasing doses of IFN-β for 24 h, followed by RNA extraction and RT-qPCR to assess IFITM mRNA expression levels. IFITM1, 2 and 3 mRNA expression was increased by IFN-β in a dose dependent manner (Fig. 1C). It appeared that the magnitude of IFITM1 induction by IFN-β treatment was higher than the levels of IFITM2 and IFITM3 induction. However, this may be due to the low basal level of endogenous IFITM1 in human keratinocytes (Fig. 1D). While differing in endogenous expression levels, IFITM1 and IFITM3 proteins were dramatically increased by IFN-β treatment (Fig. 1D).

### IFITM1 and IFITM3 Overexpression Enhances HPV16 Infectivity

**Figure 2:**
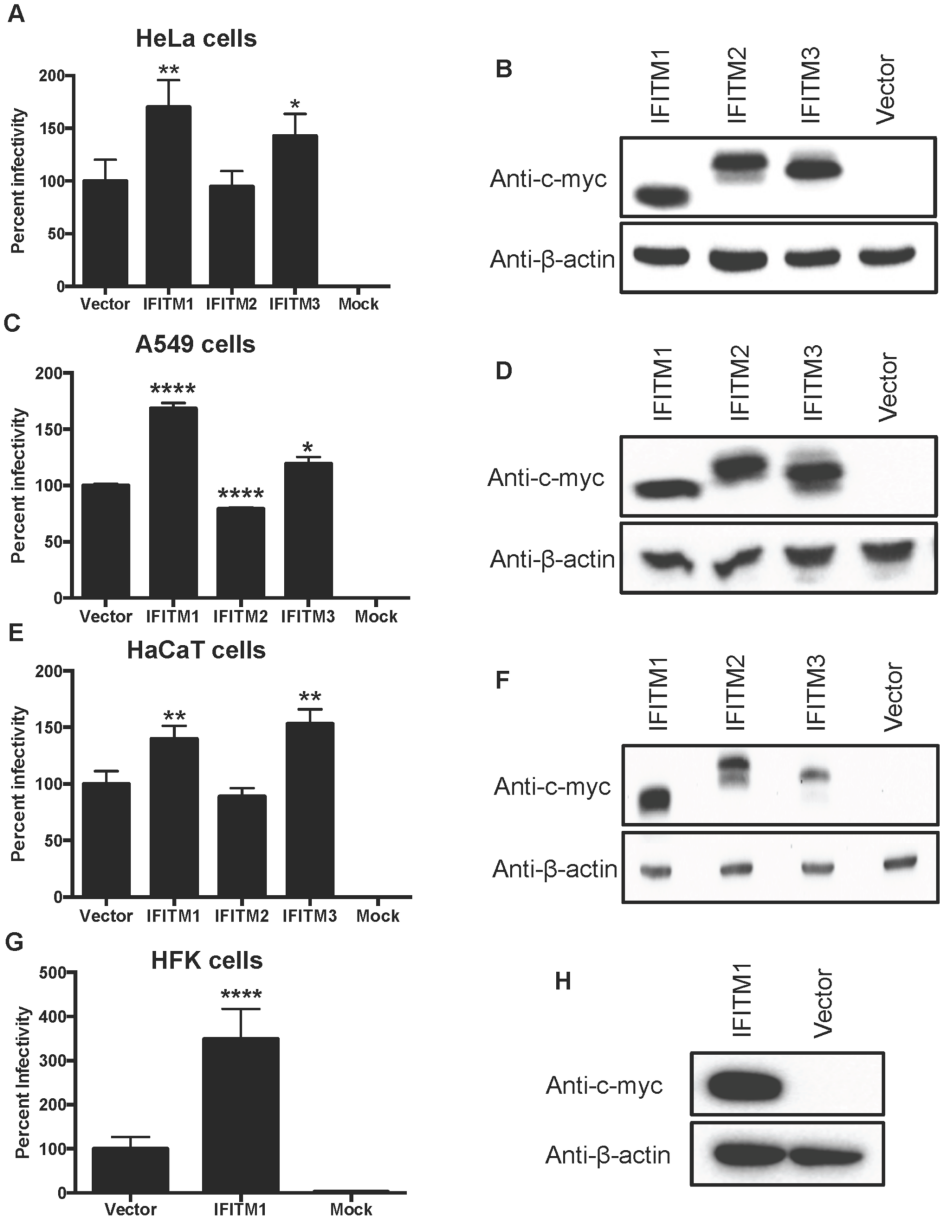
Overexpression of IFITM1 and 3 enhances HPV16 infection in epithelial cell lines and primary keratinocytes. HeLa (A & B), A549 (C & D), HaCaT (E & F), and HFK (G & H) cells expressing the indicated c-Myc-tagged IFITMs were inoculated with HPV16-LucF pseudovirions and incubated for 48 h. HPV16 infectivity was measured, as described in Fig. 1. Infectivity data was normalized to the vector alone control and shown as mean % infectivity from quadruplicate samples representative of at least two independent experiments with standard error. *P*-values were calculated as described in Fig. 1. Significant differences (*p <0.05, **p <0.01, ****p <0.0001) compared to vector transduced cells are marked with asterisks. IFITM protein expression in HeLa (B), A549 (D), HaCaT (F) and HFK (H) cells was measured by western blotting with an anti-c-Myc antibody. Detection of β-actin was used as a loading control. PageRuler plus ladder (Thermo) was used to approximate molecular weights: c-Myc-IFITM1, 18 kD; c-Myc-IFITM2 21 kD; c-Myc-IFITM3, 20 kD; β-actin, 46 kD.

To determine the effect of IFITMs on HPV16 entry, HeLa cells stably expressing c-Myc-tagged IFITM1, 2 or 3 or with vector alone (Fig. 2A–B) were infected with HPV16-LucF. At 48 hpi, infectivity was measured by luciferase assay and percent infectivity was calculated after normalization to cells transduced with the empty vector control. Despite high expression (Fig. 2B), IFITM1, 2 or 3 did not inhibit virus infection (Fig. 2A). Comparable results were obtained in A549 cells and HaCaT keratinocytes (Fig. 2C–F). Surprisingly, overexpression of IFITM1 and IFITM3 consistently enhanced HPV16 infection by 30–60% while the effects of IFITM2 were negligible (Fig. 2A, 2C and 2E). To examine the effect of IFITM1 in primary human keratinocytes, human foreskin keratinocytes (HFKs) were transduced with c-Myc-tagged IFITM1 using a retroviral delivery system [25], [41]. Consistently, IFITM1 overexpression enhanced HPV16 infection even higher at >3-fold (Fig. 2G and 2H). These results suggest that IFITM1 overexpression may facilitate HPV infection in human keratinocytes.

### IFITM1, 2 and 3 Overexpression does not Affect Infection of Ad5 or HCMV

**Figure 3:**
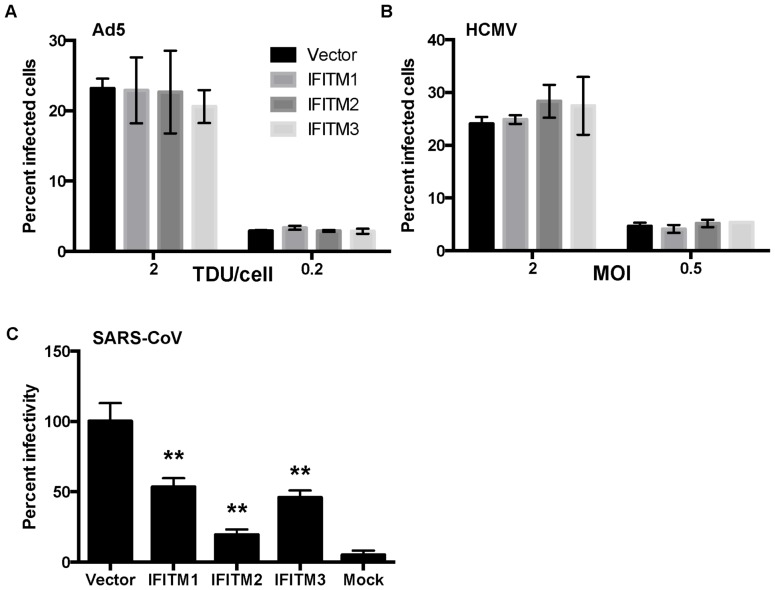
Ad5 and HCMV infections are not affected by overexpression of IFITM1, 2 and 3. HeLa cells overexpressing IFITM1, IFITM2, or IFITM3 were infected with recombinant (A) Ad5-CMV-GFP or (B) HCMV TB40E mCherry and infectivity was measured by flow cytometry at 48 hpi. Gates were set based on the uninfected control and the percentage of fluorescent cells was quantified using FlowJo software (A & B). The results are presented from at least three independent experiments with standard error. (C) HeLa cells overexpressing IFITM proteins were infected with S-protein pseudotyped SARS-CoV luciferase viruses. Infectivity was measured by Bright-Glo Luciferase Assay System (Promega), normalized to the vector alone control, and shown as percent infectivity from triplicate samples representative of two independent experiments. *P*-values were calculated as described in Fig. 1. Significant differences (**p < 0.01) compared to vector-transduced cells are marked by asterisks.

We further investigated if IFITMs restrict infection of other DNA viruses that also require acidic compartments for entry into host cells. In epithelial cells, Ad5 and HCMV entry and egress from endosomal compartments depends on low pH [45], [46]. Given that IFITM restriction is mediated along this pathway [22], [23], we hypothesized that IFITM overexpression would affect the entry processes of Ad5 and HCMV. Recombinant Ad5 and HCMV (strain TB40E) expressing GFP and mCherry, respectively, were used to infect HeLa cells stably expressing IFITM1, 2, or 3 or with vector alone. At 48 hpi, the percentage of infected cells was determined by flow cytometry. Our results showed that Ad5 and HCMV infections were unaffected by IFITM protein expression (Fig. 3A and B). It has been previously reported that S-protein mediated entry of SARS-CoV is broadly restricted by the IFITM1, 2 and 3 proteins [25]. Using a SARS-CoV S-protein pseudotyped lentivirus [39] as a positive control, we demonstrate a similar restriction of SARS-CoV infection mediated by IFITM proteins in HeLa cell lines (Fig. 3C).

### Knockdown of Endogenous IFITM1 and IFITM3 Expression does not Affect HPV16 Infectivity

**Figure 4:**
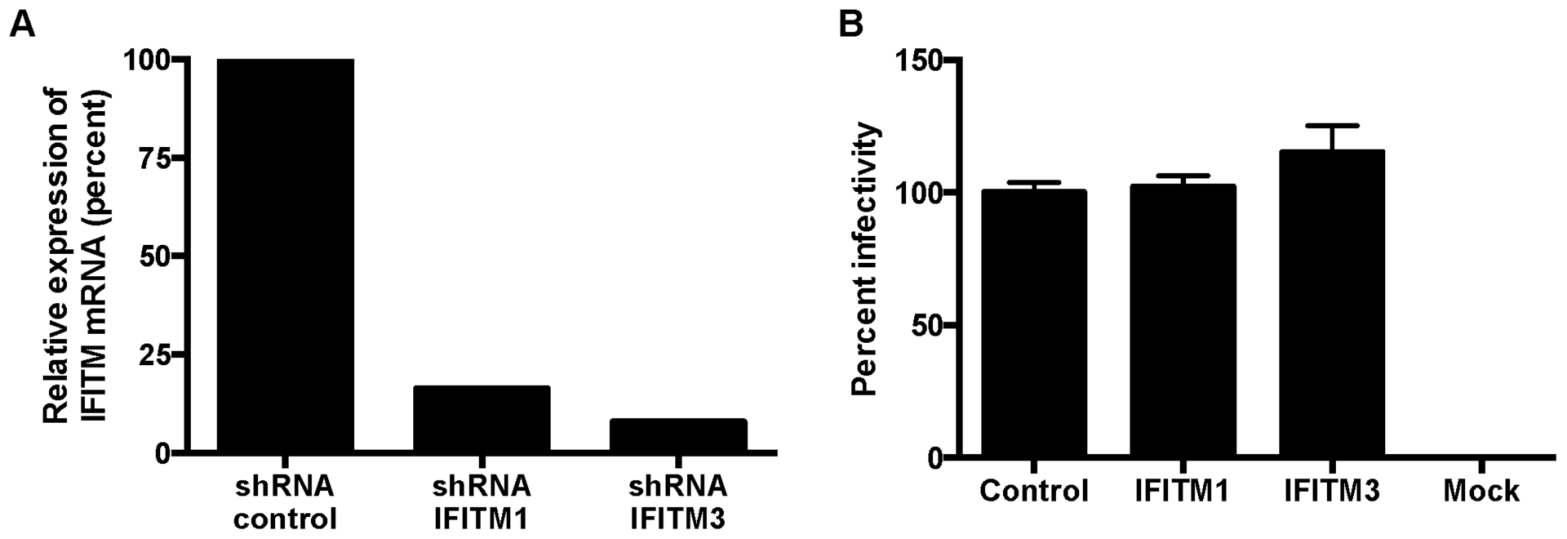
Knockdown of endogenous IFITM1 or IFITM3 does not affect HPV16 infectivity. (A) Total RNA was isolated from HeLa cells stably expressing scrambled shRNA or shRNA targeting IFITM1 or IFITM3. Expression levels of IFITM1 and IFITM3 mRNA were measured by RT-qPCR and normalized by β-actin mRNA levels. Data are presented as percent change in mRNA expression of knockdown cells compared to cells with scrambled shRNA. (B) Cells in (A) were infected with HPV16-LucF and analyzed as described in Figure 1.

To determine whether endogenous IFITM expression affects HPV entry, IFITM1 or IFITM3 was knocked down in HeLa cells by stable expression of IFITM1, IFITM3 or control scrambled shRNA [25]. Since IFITM2 expression appeared to have no effect on HPV16 infectivity, we focused only on IFITM1 and IFITM3. The levels of target gene knockdown were determined by RT-qPCR (Fig. 4A). Although IFITM1 and IFITM3 show some sequence homology, no off target effects were noted by shRNA knockdown (Fig. S2). Interestingly, knockdown of IFITM1 or IFITM3 expression had no effect on HPV16-LucF infection (Fig. 4B). Taken together, our results suggest that, compared to RNA viruses, the DNA viruses HPV, HCMV, and Ad5 might rely on different entry mechanisms to avoid IFITM restriction.

### IFITM1 Overexpression Delays HPV Capsid Protein Degradation

**Figure 5:**
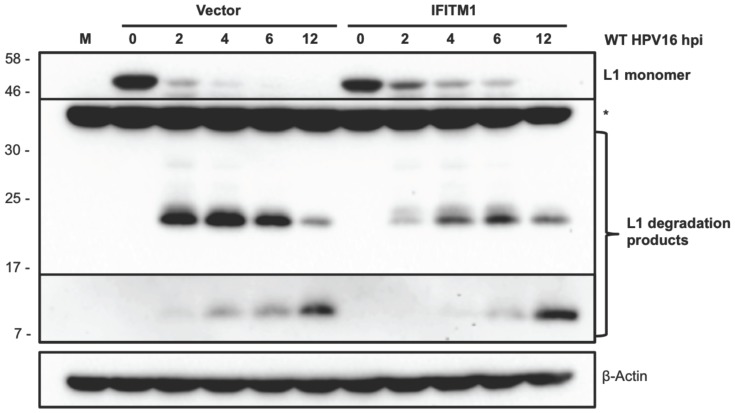
IFITM1 overexpression delays degradation of HPV16 L1 capsid protein in primary keratinocytes. HFKs were infected with 10,000 vge/cell of HPV16 virions and placed at 4°C for 1 h to inhibit endocytosis. Unbound virions were washed away and cells were incubated at 37°C for the indicated time points. Cells were trypsinized to remove non-internalized virions, and then lysed to detect internalized virions by western blotting for L1 capsid protein. Detection of β-actin was used as a loading control. Boxed bands are from a longer exposure. M, mock. *Non-specific band.

Previous studies show that overexpression of IFITM3 disrupts the composition of endosomal compartments [36], [47] and influences the rate of reovirus capsid protein degradation [29]. Given the consistent effects of IFITM1 overexpression on HPV16 infection in primary keratinocytes, we determined whether IFITM1 altered the kinetics of HPV16 L1 major capsid protein degradation. HFKs were inoculated with HPV16 virions containing full-length viral genomes for 1 h at 4°C to allow for virus attachment but not internalization. Internalization was initiated by shifting the cells to 37°C for 0, 2, 4, 6 and 12 h. At the indicated time points, the cells were trypsinized to remove non-internalized virus, then harvested for HPV16 L1 western blotting. Intriguingly, HFKs overexpressing IFITM1 exhibited delayed L1 degradation compared to the empty vector control, showing reduced accumulation of the 22 and 12 kD degradation products as early as 2 hpi (Fig. 5). Interestingly, we previously reported that the autophagy inhibitor, 3-methyladenine (3-MA), delays the degradation of HPV16 L1 capsid proteins during entry in a strikingly similar manner as IFITM1 overexpression [41]. Further, 3-MA also significantly enhanced HPV16 infection in HFKs [41]. As Feeley et al. demonstrated a role for IFITM3 in autophagosome-lysosome fusion during influenza A virus infection [36], our results imply that IFITM1 overexpression may alter cellular compartments in a similar manner that partially protects the HPV capsid from degradation in lysosomes and autolysosomes.

## Discussion

It is well known that innate immune responses are critical for protecting host cells from early establishment of virus infection. Following recognition of viral components by pattern recognition receptors, type I IFN production is induced which triggers localized antiviral activities through various ISGs [48]. Here we report the role of type I IFNs, IFN-α and IFN-β, in restricting HPV entry into human keratinocytes. As shown in Figure 1, IFN-β significantly inhibited HPV infection at much lower concentrations than IFN-α, while IFN-α efficiently induced ISGs in human keratinocytes. One explanation for these differential effects is that IFN-α and -β induce different groups of ISGs. Microarray analysis of cells treated with either IFN-α or IFN-β identified more than 20 candidate genes whose mRNA levels are selectively induced by IFN-β but not IFN-α. These most notably include PKR, ISG-56 (IFIT1), ISG-58 (IFIT5), and HIF-1α, while the remaining genes lack annotated antiviral activity [49]. An unbiased analysis of genes preferentially induced by IFN-β in keratinocytes may be useful in identifying candidate host factors that restrict HPV entry and replication. Alternatively, the differences in HPV infectivity may be due to differences in interruption of cell proliferation triggered after IFN exposure. IFN-β exhibits strong anti-proliferative effects on human cancer cells at concentrations far lower than those for IFN-α [50]. As we previously revealed that HPV requires host cell mitosis for virus entry [43], disrupting cell proliferation by IFN-β may interfere with HPV infection.

IFITM proteins inhibit several RNA viruses that rely on low-pH in late endosomes or lysosomes for virus entry [22], [23]. It is well established that HPV requires these acidified compartments for uncoating and egress from the endosomal pathway [33]–[35], thus IFITMs would serve as an attractive candidate for HPV restriction. Using various epithelial cell lines and primary keratinocytes expressing IFITMs, we show that HPV infection is surprisingly enhanced by IFITM1 and IFITM3 overexpression (Fig. 2). A similar enhancement has been observed with pseudovirions expressing the entry proteins of several arenaviruses and a retrovirus, Moloney murine leukemia virus (MMLV) [24]. Thus, our and previous results [24] suggest that resistant viruses may take advantage of endocytic trafficking altered by overexpression of IFITM. Additionally, we demonstrate that IFITM1, 2 and 3 did not inhibit infection of two other DNA viruses, Ad5 and HCMV, whereas infection of SARS-CoV, a positive control, was broadly restricted (Fig. 3). The low pH of the endocytic pathway is required for adenovirus infection [51], [52]. However, it has been reported that adenovirus egress from endosomes is rapid and likely mediated by penetration in early endosomes but not late endosomes [45], [53]. Entry mechanisms of HCMV vary depending on different virus strains and the host cell types [54]. While HCMV entry into fibroblasts is mediated by pH-independent fusion at the host cell plasma membrane [55], virus entry into epithelial cells requires endocytosis and pH dependent fusion within endosomes [46]. Because we utilized an epithelial cell model (HeLa), HCMV entry might occur along the endocytic pathway, although the exact point of egress has still yet to be determined. Nevertheless, the tested DNA viruses, HPV, HCMV, and Ad5, similarly require endocytosis and low pH for entry into host cells, and are not inhibited by IFITMs. Further comparative studies of larger panels of DNA viruses, with various entry mechanisms, might aid in defining the role of IFITMs during DNA virus infection and clarify the differential routes of endosome trafficking in RNA and DNA virus entry into host cells.

Our results provide the first evidence that IFITMs do not restrict and potentially enhance infection of DNA viruses (Fig. 2). Overexpression of IFITM1 delayed L1 capsid protein degradation in primary keratinocytes (Fig. 5), which may explain an enhancement of virus infection by IFITM1. Further investigations into how viruses bypass IFITM restriction may provide more detailed mechanistic insights into virus trafficking and help clarify productive routes of infectious entry. Taken together, our results suggest that HPV and other DNA viruses may have evolved to avoid IFITM1, 2 and 3 restriction during entry into host cells.

## Supporting Information

Figure S1 **IFN-α treatment stimulates the induction of ISGs in keratinocytes in a dose dependent manner.** HaCaT cells were treated with 70 or 350 U/mL IFN-α for 24 h. Total RNA was extracted and expression levels of the indicated ISGs were measured by RT-qPCR. Data is presented as fold change relative to β-actin mRNA, followed by normalization to PBS-treated cells. Error bars represent SEM.
(TIF)

Figure S2 **IFITM knockdown in HeLa cells is specific.** Total RNA was isolated from HeLa cells stably expressing scrambled shRNA or shRNA targeting IFITM1 or IFITM3. Expression levels of IFITM1 and IFITM3 mRNA were measured by RT-qPCR and normalized by β-actin mRNA levels. Data is presented as % change in mRNA expression of knockdown cells compared to cells with scrambled shRNA.
(TIF)

Table S1 **List of primers used for RT-qPCR.**
(PDF)
