# Supplementary figures and images for: The Antiviral Restriction Factors IFITM1, 2 and 3 Do Not Inhibit Infection of Human Papillomavirus, Cytomegalovirus and Adenovirus
Source: PLoS One. 2014 May 14;9(5):e96579. doi: 10.1371/journal.pone.0096579 (PMC4020762; doi:10.1371/journal.pone.0096579)

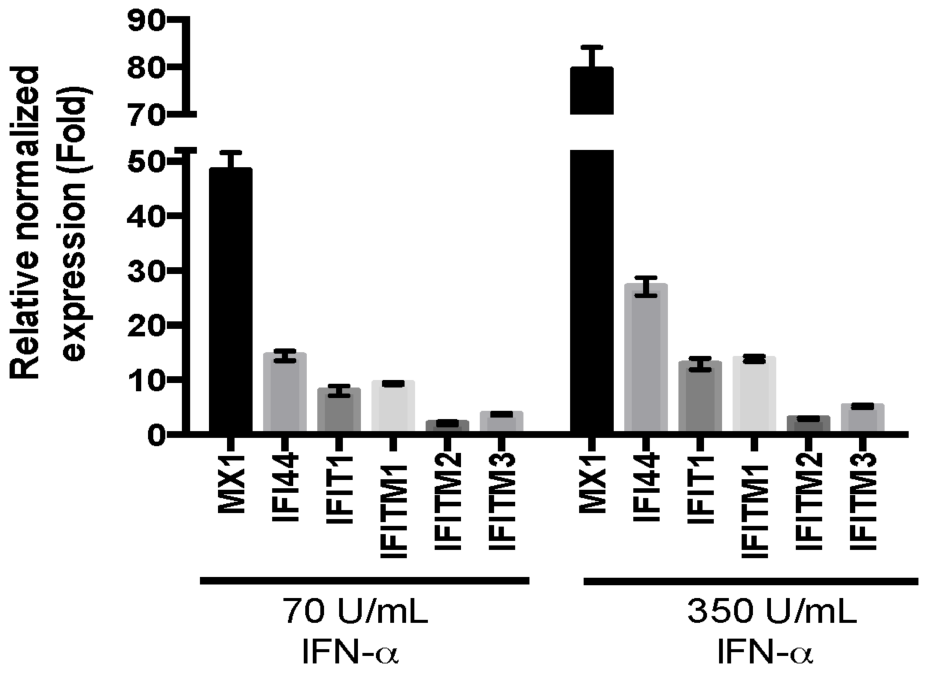

Supplement: Figure S1 — IFN-α treatment stimulates the induction of ISGs in keratinocytes in a dose dependent manner. HaCaT cells were treated with 70 or 350 U/mL IFN-α for 24 h. Total RNA was extracted and expression levels of the indicated ISGs were measured by RT-qPCR. Data is presented as fold change relative to β-actin mRNA, followed by normalization to PBS-treated cells. Error bars represent SEM. (TIF) [file pone.0096579.s001.tif]

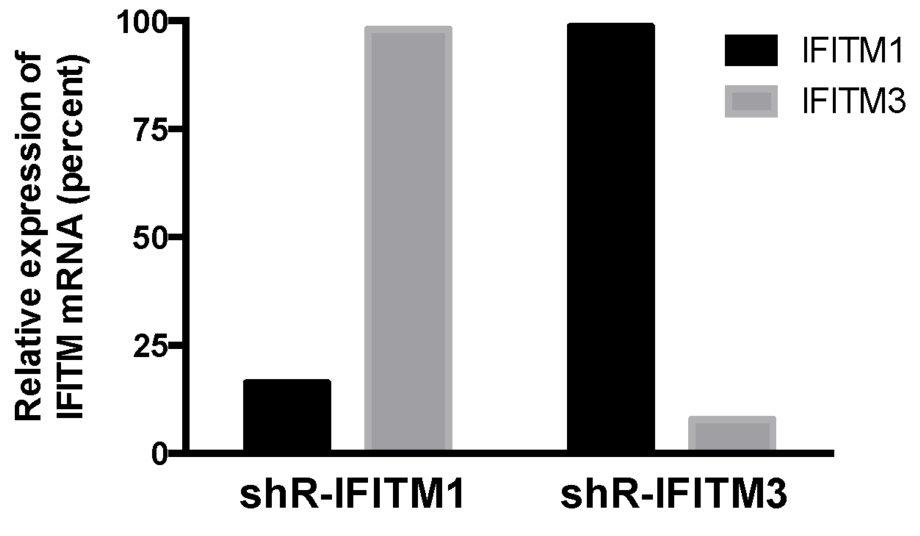

Supplement: Figure S2 — IFITM knockdown in HeLa cells is specific. Total RNA was isolated from HeLa cells stably expressing scrambled shRNA or shRNA targeting IFITM1 or IFITM3. Expression levels of IFITM1 and IFITM3 mRNA were measured by RT-qPCR and normalized by β-actin mRNA levels. Data is presented as % change in mRNA expression of knockdown cells compared to cells with scrambled shRNA. (TIF) [file pone.0096579.s002.tif]
