# Supplementary material for: The Antiviral Restriction Factors IFITM1, 2 and 3 Do Not Inhibit Infection of Human Papillomavirus, Cytomegalovirus and Adenovirus
Source: PLoS One. 2014 May 14;9(5):e96579. doi: 10.1371/journal.pone.0096579 (PMC4020762; doi:10.1371/journal.pone.0096579)
Supplement: Table S1 — List of primers used for RT-qPCR. (PDF) [file pone.0096579.s003.pdf]

Supplementary Table 1

Table S1. Primers used in this study

Gene	GenBank Accession	Forward (5' - 3')	Reverse (5'-3')
IFITM1	NM_003641.3	ACTCCGTGAAGTCTAGGGACA	TGTCACAGAGCCGAATACCAG
IFITM2	NM_006435.2	ATCCCGGTAACCCGATCAC	CTTCCTGTCCCTAGACTTCAC
IFITM3	NM_021034.2	GGTCTTCGCTGGACACCAT	TGTCCCTAGACTTCACGGAGTA
MX1	NM_001282920.1	TTTTCAAGAAGGAGGCCAGCAA	TCAGGAACTTCCGCTTGTCG
IFIT1	NM_001270930.1	GAAAGCCTCAGTCTTGCAGC	CATCACCATTTGTACAAGAGCCT
IFI44	NM_006417.4	TGTGAGGTCCAAGCTAGAGGA	CCATAGCATTCGTCTCAGAGC
